# Based on regional heterogeneity: measurement of hospital service efficiency and analysis of spatial effects

**DOI:** 10.3389/fpubh.2025.1556737

**Published:** 2025-04-03

**Authors:** Qianwen Song, Anyu Ye, Mengting Zhou, Ting Chen, Wenlian Jiang

**Affiliations:** ^1^Chengdu Shuangliu District Maternal and Child Health Hospital, Chengdu, China; ^2^School of Pharmaceutical Economics and Management, Anhui University of Chinese Medicine, Hefei, China; ^3^School of Nursing, Anhui University of Chinese Medicine, Hefei, China; ^4^School of Pharmacy, Anhui University of Chinese Medicine, Hefei, China

**Keywords:** hospital service efficiency, technological heterogeneity, meta-frontier SBM, spatial Durbin model, regional differences

## Abstract

**Background:**

Hospital service efficiency is a vital indicator of the effectiveness of a country’s healthcare system. If regional heterogeneity is ignored in measuring the efficiency of health services, erroneous judgments may result.

**Methods:**

This study utilized the Meta-Frontier Slack-Based Measure (SBM) model to evaluate the efficiency of hospital services across various Chinese provinces from 2009 to 2022. Efficiency comparisons were made using regional and common frontiers, while the technical gap and its decomposition index were applied to identify the sources of efficiency disparities among regions. The Spatial Durbin Model was then employed to analyze the spatial spillover effects of hospital service efficiency.

**Results:**

The findings reveal that under the common frontier, the Central South regions demonstrate the highest efficiency, averaging 0.7549, followed by East China at 0.7184, the Southwest at 0.6245, the Northwest at 0.5497, North China at 0.4884, and the Northeast at 0.3571. Technical disparities among China’s regional hospital services form three distinct tiers: the first tier includes Central South China, the second tier comprises the Southwest, Northwest, and North China, and the third tier is the Northeast. Management inefficiency predominantly affects the first tier, whereas both management and technical inefficiencies impact the second and third tiers. Furthermore, hospital service efficiency has significant spatial spillover effects, notably enhancing the efficiency of neighboring provinces.

**Conclusion:**

To enhance healthcare service efficiency, it is imperative to implement precise identification of technological and managerial deficiencies, coupled with the formulation of long-term strategic plans adaptive to demographic shifts and evolving healthcare demands, while simultaneously strengthening crisis management capabilities to ensure the stability and sustainability of service efficacy.

## 1 Introduction

Hospitals play a pivotal role in a country’s public health service system. Their development is essential for optimizing this system, ensuring equity, controlling the exponential growth of medical expenses, and enhancing residents’ health standards. However, hospitals are also the most costly component, accounting for 50–80% of total health expenditures (1). The efficiency of hospital services directly influences the utilization of the medical and health system. According to the World Health Organization (WHO), 20–40% of resources in health care systems are underutilized, with a higher percentage in low-income countries (2). In China, over the past few decades, the issue of medical and health efficiency has been particularly pronounced. Factors such as economic growth, population expansion, rapid aging, and improved personal health awareness have led to a surge in demand for medical and health services. In response to these challenges, China initiated a new round of healthcare system reform in 2009. This reform has led to significant progress, including rapid growth in China’s medical and health sector, a continuous increase in the total amount of medical resources, and a substantial expansion in the coverage of health services. However, it has also introduced new issues. Economic disparities among regions have become more pronounced due to geographical and spatial distribution differences, resulting in significant disparities in the allocation of health resources between regions and between urban and rural areas (3–5). Additionally, differences in technical levels and production management methods across regions have further exacerbated disparities in health service efficiency. These differences not only limit the public’s access to high-quality and equitable medical services but also threaten economic development and social stability (6). Therefore, an urgent task for China’s medical and health system is to enhance the overall efficiency of hospital services, address the challenges faced by hospitals in various regions, ensure balanced development of health services across regions, and meet the needs of public health services.

Currently, the academic community has conducted extensive research aimed at enhancing the efficiency of hospital services in China. This research encompasses the measurement, influencing factors, and impact of hospital services. Most studies employ statistical techniques such as Data Envelopment Analysis (DEA) and Stochastic Frontier Analysis (SFA) to evaluate the efficiency of hospital services throughout China (7–9). These studies indicate disparities in service efficiency within China’s healthcare system at both the national and provincial levels. Researchers like Jiang et al. (10) propose that these disparities primarily result from the uneven distribution of health resources. Additionally, some scholars have utilized the Malmquist model to analyze China’s health efficiency (11). Factors such as economic development, health investment intensity, and urbanization level have been identified in the literature as significant influences on hospital service efficiency (12–14). In terms of spatial effects on hospital service efficiency, methods like Moran’s I and *β* convergence models are commonly applied. From a spatial perspective, these studies examined whether spatial correlation exists in China’s hospital service efficiency, identified the clustering characteristics of its spatial distribution, and investigated the presence of a spatial ‘catching-up effect’ in the efficiency of hospital services across different regions (15, 16). However, existing research often focuses on the national level, individual provinces, or the three major regions, which, while informative, tends to overlook the regional nuances of China’s healthcare system (17, 18). This approach can lead to a generalized view that does not capture the full scope of medical services provided. There is a need for more nuanced analysis to account for regional variations in technical capabilities and efficiency imbalances.

Secondly, most studies have used different SFA and DEA modeling approaches to study efficiency, but each of these models has certain limitations. The SFA model is suitable for parametric analysis in a single technology setting, but it can only handle a single output, making it difficult to address the reality of multiple inputs and outputs in health systems. While the DEA model effectively addresses the limitation of the SFA approach by handling multiple outputs, it requires inputs and outputs to vary in strict proportion, whereas in practice, inputs and outputs often have slacks that are not strictly proportional. In contrast, the SBM model allows for simultaneous consideration of input and output slacks without requiring strictly proportional changes in inputs and outputs, making it closer to the real production process. By fitting the real production process more accurately, the SBM model is able to distinguish between input and output efficiencies or inefficiencies more effectively. Research has indicated significant differences in hospital service efficiency across China’s regions (19), however, these studies usually use economic zones as the dividing criteria, such as the three major economic regions in the east, center, and west. This approach is too general, as there are significant disparities in the level of economic development and resource endowment between these zones, as well as substantial technological gaps in the health services that each zone specializes in. Therefore, provinces cannot simply be categorized as one region based solely on geographical attributes. Ignoring the technological disparities between these regions can result in overly simplistic research findings, hindering the identification of the actual causes of efficiency loss in hospital services. While advancements have been made in understanding the spatial effects on health service efficiency, there is a notable lack of analysis on how spatial spillover effects specifically influence China’s health service efficiency. Additionally, there is an absence of quantitative assessment regarding the impact of spatial effects on the efficiency of health services.

Overall, current research on hospital services in China has focused on assessing service efficiency levels, regional disparities, and the existence of spatial effects. However, these studies often neglect a detailed analysis of the causes of inefficiency and the mechanisms behind spatial spillover effects. To address this gap, this study employs the meta-frontier approach along with the non-radial Slack-Based Measure model to analyze hospital service efficiency, technological disparities, and their structural breakdowns across China’s six major regions. Additionally, the Spatial Durbin Model is utilized to investigate the spatial spillover effects of hospital service efficiency.

The potential marginal contributions of this study are as follows: (1) It expands the scope of hospital service efficiency research from the traditionally examined three major regions to six major regions, providing a deeper analysis of the evolving trends and technological disparities in hospital service efficiency across various Chinese regions. (2) Methodologically, in response to the limitations of the non-expected Slack-Based Measure model due to technological heterogeneity, the study incorporates a meta-frontier-based non-expected SBM model for more precise measurement and optimization. (3) Recognizing the spatial variations in hospital service efficiency across different regions, the study also utilizes the Spatial Durbin Model to examine the associated spatial spillover effects and the factors influencing them.

## 2 Methods and data sources

### 2.1 Indicator selection

#### 2.1.1 Selection of hospital service efficiency indicators

Labor and capital are the two most extensively utilized input indicators in research on medical institution service efficiency (20, 21). For capital inputs, this study draws upon previous research by selecting the number of hospitals and beds as input variables to represent capital investment (22). Regarding labor inputs, following the method of Jinjiang et al. (23), this paper uses the input of health technicians in hospitals to indicate labor investment. Specifically, the number of hospital health technicians, the number of beds, and the number of hospitals directly reflect the allocation of medical resources and service capacity. Their adequacy and rational allocation form the basis for improving the efficiency of medical services. Furthermore, the volume of outpatient visits, inpatient visits, and bed utilization rate are crucial indicators for assessing the utilization and influencing factors of medical services (24). This research measures hospital health service output by examining the number of outpatient visits, inpatient visits, and bed utilization rate in hospitals. Specifically, bed occupancy rates, outpatient visits, and hospital discharges reflect the operational effectiveness and service capacity of healthcare organizations. Efficient outputs in these areas indicate that resources are being used effectively. Additionally, the incidence and mortality rates of infectious diseases serve as indicators of the effectiveness of public health services in disease prevention and control within a region (25). This study focuses on the mortality rates of category A and B infectious diseases as measures of disease prevention and control. Specifically, the mortality rate for A and B diseases represents a negative indicator of the quality of services, which directly affects the overall efficiency assessment. A low mortality rate not only indicates a good medical outcome but also suggests a higher quality of services. Specific indicators were selected as shown in Table 1.

**Table 1:** Indicators of efficiency of hospital services.

Type	Indicator
Input	Number of Hospitals
	Number of Health Technicians
	Number of Hospital Beds
Desired output	Total Outpatient Visits
	Total Hospital Discharges
Undesired output	Mortality Rate for Diseases A and B

#### 2.1.2 Control variable indicator selection

As shown in Table 2, level of human capital (EDU) reflects the level of education, which can improve residents’ health literacy and enhance the professional skills of doctors, thereby impacting the efficiency of healthcare services.

**Table 2:** Definition and descriptive statistics of control variables.

Variable	Variable definition	Observes	Maximum value	Minimum value	Standard deviation	Mean
EDU	Number of students enrolled in higher education/total population (%)	434	0.0436	0.0079	0.0061	0.0201
OPEN	Total import/export trade/GDP (%)	434	1.4638	0.0076	0.2913	0.2710
LHE	Government health expenditure/total government expenditure (%)	434	0.1393	0.0397	0.0163	0.0767
LST	Government expenditure on science and technology/total government expenditure (%)	434	0.0720	0.0030	0.0152	0.0207
ECON	Indicators of total GDP	434	11.7715	6.0996	1.0408	9.6683

OPEN indicates the degree of economic openness, which may be related to the cross-border demand for healthcare services, international cooperation, and the introduction of advanced medical technologies.

Level of government investment in health (LHE) reflects the government’s prioritization of the healthcare sector. A higher LHE ratio typically means that the government prioritizes healthcare in its fiscal budget, which directly influences the funding available to the healthcare industry. Adequate financial support can improve healthcare infrastructure, enhance staff training, and introduce advanced technologies, thereby increasing the overall efficiency and quality of healthcare services.

Level of government investment in science and technology (LHT) indicates the capacity for technological innovation in a region. Technological innovation can drive advancements in medical technology, thereby increasing the efficiency of healthcare services.

GDP represents the overall economic level and per capita income, which are crucial indicators of regional healthcare demand and resource allocation. A higher GDP suggests greater economic resources and potential for investing in healthcare infrastructure, thus influencing the efficiency and quality of healthcare services.

### 2.2 Data sources

**Figure 1:**
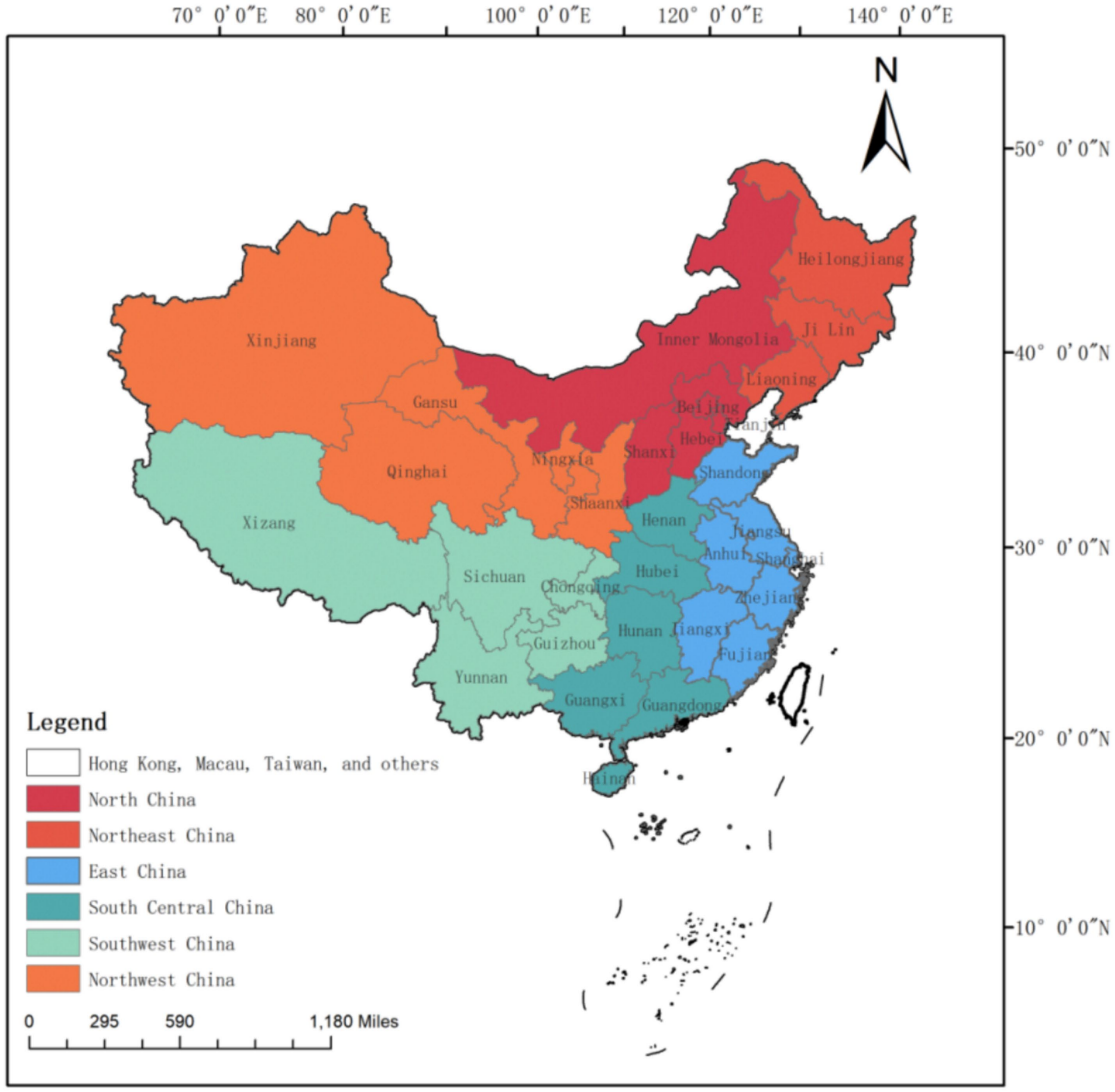
Map of China’s six major regions.

The data used to measure medical service efficiency indicators are sourced from the China Health Statistical Yearbook (2010–2023), while data for relevant control variables are obtained from the China Statistical Yearbook (2010–2023). For regional classification, this research references the six major administrative regions established in the early years of the People’s Republic of China, categorizing them into the Northeast, North China, East China, Central South China, Northwest, and Southwest regions (see Figure 1 for specifics). This system of six administrative regions is also believed to have formed the basis for China’s economic and higher education structures since its establishment (26, 27).

### 2.3 Methods

#### 2.3.1 Meta-frontier SBM

Given the technological diversity among China’s provincial healthcare systems, it is inevitable that each province encounters its unique production frontier. Using a blanket sample for efficiency assessment would fail to accurately capture the realities of individual provinces. Therefore, this study draws on the work of Zhang et al. (28) to develop a Meta-Frontier Slack-Based Measure model for evaluating hospital service efficiency across China’s 31 provinces. The SBM model is particularly adept at addressing slack in input and output variables, effectively handling efficiency assessments in the presence of undesirable outputs, and distinguishing efficiency variations among effective decision-making units. Additionally, since traditional efficiency measurement methods rely on contemporaneous frontiers and do not permit comparisons across different time periods, the study adopts the concept of a global production technology set as proposed by OH (29), culminating in the construction of a Meta-Frontier global SBM model (30). The specific approach involves constructing both a common frontier production possibility set and group-specific frontier production possibility sets. These are then applied to the SBM model to calculate the common frontier efficiency and the efficiency of each group frontier.

#### 2.3.2 Construction of the undesirable SBM model

This paper adopts an SBM model that includes undesirable outputs to construct efficiency measurement models under both the common frontier and group frontier (18), which can be expressed as follows:


$$\rho =\min\frac{1-\frac{1}{M}\sum_{m=1}^{M}\frac{s_{m0}^{x}}{x_{m0}}}{1+\frac{1}{R+J}\left(\sum_{r=1}^{R}\frac{s_{r0}^{y}}{y_{r0}}+\sum_{j=1}^{J}\frac{s_{j0}^{b}}{b_{j0}}\right)}$$

(1) $$s.t.\;\overset{H}{\underset{h=1}{\sum }}\overset{N^{h}}{\underset{n=1}{\sum }}\lambda_{n}x_{mn}+s_{m0}^{x}=x_{m0}$$

$$\overset{H}{\underset{h=1}{\sum }}\overset{N^{h}}{\underset{n=1}{\sum }}\lambda_{n}y_{rn}-s_{r0}^{y}=y_{r0}$$

$$\overset{H}{\underset{h=1}{\sum }}\overset{N^{h}}{\underset{n=1}{\sum }}\lambda_{n}b_{jn}+s_{j0}^{b}=b_{j0}$$

$$\lambda_{n}^{\mathrm{meta}},s_{m0}^{x},s_{r0}^{y},s_{j0}^{b}\geq 0$$

Where $s_{m0}^{x}$ , $s_{r0}^{y}$ , $s_{j0}^{b}$ represent the slack variables for inputs, desired outputs, and unintended outputs, respectively. A decision-making unit is efficient if and only if $s_{m0}^{x}$ *= 0*, $s_{r0}^{y}$ *= 0*, $s_{j0}^{b}$ *= 0*, which means *ρ* = 1. When ρ < 1, it indicates that the decision-making unit is below the frontier and can reach an efficient frontier by reducing inputs, decreasing unintended outputs, or increasing desired outputs (31).

#### 2.3.3 Constructing the common frontier and group production possibility sets

Define the technology set $T^{u}$ for a common DMU, which can be expressed as:


(2) $$T^{u}=\left\{\left(x,y,b\right)|\;x\geq 0,\;y\geq 0,\;b\geq 0;\;x\;\mathrm{can}\;\mathrm{produce}\;\left(y,\;b\right)\right\}$$

where u is the type of technology ensemble and $T^{u}$ represents the level of inputs and technology to obtain the output $P^{u}\left(y,b\right)$ .

The production possibility set for the common DMU is:


(3) $$p^{\mathrm{meta}}\left(x\right)=\left\{\left(y,b\right)|\left(x,y,b\right)\in T^{u}\right\}$$

The expression for the common distance function considering undesirable outputs, denoted as $D^{u}$ , is:


(4) Duxyb=infθθ>0:yθ∈Tu=MTExyb

For group g, the expression for the distance function considering undesirable outputs, denoted as $D^{g}$ , is:


(5) Dgxyb=infθθ>0:yθ∈Tu=GTEkxyb,k∈1,2,3,4,5,6

Here, these functions express radial distances. $MTE\left(x,y,b\right)$ and ${GTE}^{k}\left(x,y,b\right)$ represent the efficiency values under the common frontier and group frontier, respectively.

These values can be obtained by applying the distance functions $D^{u}$ and $D^{g}$ in the Equation 1 under the common frontier and group frontier settings.

#### 2.3.4 Technology gap ratio (TGR)

TGR, or the Technological Gap Ratio, typically reflects the technological gap between the common frontier and the group frontier. A higher TGR value indicates that the actual production technology level is closer to the potential production technology level, implying that the decision-making unit is operating more efficiently relative to its potential. Conversely, a lower TGR value suggests that the gap between the decision-making unit’s production technology level and the potential production technology level is widening, indicating inefficiency. It is specifically expressed as:


(6) $$0\leq TGR=\frac{D^{u}\left(x,y,b\right)}{D^{g}\left(x,y,b\right)}=\frac{MTE\left(x,y,b\right)}{GTE^{k}\left(x,y,b\right)}\leq 1$$

Although the TGR can help analyze the gap between each province’s hospital service efficiency and the potential optimal governance efficiency, it does not identify the root causes of inefficiency in hospital services within each province. To address this limitation, and drawing on the research by Chiu et al. (32) and Choi et al. (33), this study decomposes the inefficiency value, known as the inefficiency value of hospital services (MGEI), of hospital services in each province under the common frontier, as shown in Equations 7–9.


(7) MI=1−GTE

(8) $$TGI=MTE\times \left(1-TGR\right)$$

(9) MGEI=MGI+TGI=1−MTE

In this context, MGI (Management Gap Index) represents the inefficiency arising from the low management level of each province when facing the same group frontier. Meanwhile, TGI (Technological Gap Index) indicates the technical inefficiency resulting from the disparity between the actual production technology and the common frontier production technology (34).

#### 2.3.5 Spatial Durbin model

Considering that hospital service efficiency in different provinces in China is influenced not only by the medical service level and social factors within each province but also by the flow of capital, human resources, and technology, which can both affect and be affected by other provinces (35), there is likely a correlation in the spatial distribution of hospital service efficiency. Therefore, this study introduces spatial econometric models to capture the spillover effects of the research sample over time and space, and examines the spatial lag term of the dependent variable (36).

The model setup is as follows:


(10) $$MTE_{\mathrm{it}}=\alpha_{0}+\rho W_{n}MTE_{\mathrm{it}}+\beta_{1}{Ζ}_{\mathrm{it}}+\beta_{2}W_{n}{Ζ}_{\mathrm{it}}+\varepsilon_{\mathrm{it}}$$

In Equation (10), ${MTE}_{\mathrm{it}}$ represents the hospital service efficiency of province i in year t, $W_{n}$ is the spatial weight matrix, and $W_{n}{MTE}_{\mathrm{it}}$ is the spatial lag term of the hospital service efficiency for that province.

Z_it_ represents the control variables, mainly including educational level (EDU), level of openness to the outside world (OPEN), level of health expenditure (LHE), level of science and technology expenditure (LST), and level of economic development (ECON) (37–39). The definitions and data sources for these variables are shown in Table 1. W_n_Z_it_ is the spatial lag term of the control variables. $\varepsilon_{\mathrm{it}}$ denotes the error term, and $\varepsilon_{\mathrm{it}}∼N\left(0,\sigma_{\mathrm{it}}^{2}\right)$ , ρ and $\beta_{2}$ are the spatial autocorrelation coefficients.

## 3 Results

### 3.1 Efficiency analysis

Using the hospital service efficiency indicator system developed in the previous section, along with the meta-frontier theory (Equations 2–5) and the SBM model (Equation 1), we assess the efficiency of hospital services across China’s 31 provinces. This analysis involves evaluating efficiency levels under both common frontier and group frontier scenarios.

The results, presented in Table 3, indicate that common frontier efficiency and group frontier efficiency represent the distance function values calculated using the common and group boundaries as benchmarks. This reflects how far the actual output of a set of production units falls short of the potential output at both boundaries, given the same level of input (40). From the standpoint of technological diversity, this research examines the average differences across regions. From a national perspective, the mean efficiencies under the common frontier and group frontier frameworks are 0.6111 and 0.8333, respectively. This suggests a potential for a 38.89% improvement when compared to the nation’s best technology and a 16.67% improvement compared to the regional best technology. The significant difference between the common and group frontiers primarily stems from these methods being based on different sets of production technologies.

**Table 3:** Descriptive statistics of regional hospital service efficiency under common and group frontiers.

Region	Common frontier efficiency				Group frontier efficiency			
	Mean	Maximum	Minimum	SD	Mean	Maximum	Minimum	SD
Nationwide	0.6111	0.9381	0.2863	0.177	0.8333	0.9591	0.4685	0.1207
North China	0.4884	0.6788	0.2863	0.1482	0.7392	0.9072	0.4685	0.1927
Northeast China	0.3571	0.3925	0.3122	0.0335	0.886	0.9436	0.7827	0.0733
East China	0.7184	0.9344	0.5597	0.1187	0.8075	0.947	0.6406	0.0937
Central South	0.7549	0.9381	0.5735	0.1208	0.8425	0.9576	0.6334	0.1119
Southwest China	0.6245	0.7846	0.4531	0.1358	0.8857	0.9538	0.7698	0.0677
Northwest China	0.5497	0.7843	0.4348	0.1313	0.8687	0.9591	0.8079	0.0507

Regionally, the Central South regions exhibit the highest average hospital service efficiency at 0.7549, followed by the eastern region at 0.7187 and the southwestern region at 0.6245. In contrast, the northern, northeastern, and northwestern regions fall below the national average, with averages of 0.4884, 0.3571, and 0.3571, respectively. This indicates substantial room for improvement in hospital service efficiency across China. The Central South regions, with their advantages in talent, capital, and technology, are better positioned to enhance hospital service efficiency. The lower overall service efficiency in the other four regions suggests these areas are not effectively utilizing their health resources and need to strengthen their health service capabilities. This is primarily because these regions are distant from China’s core economic areas, receive less economic momentum, and are strongly influenced by the talent and capital attraction of the economically advanced provinces in the eastern and central southern regions. This has led to an outflow of health resources and patients, thereby reducing hospital service efficiency.

Additionally, the group frontier results indicate the average hospital service efficiency across different regions, in descending order, is as follows: northeastern, southwestern, northwestern, central south, eastern, and northern regions. These regions have potential efficiency improvements of 11.40, 11.43, 13.13, 15.75, 19.25, and 26.08%, respectively. When comparing the common and group frontier efficiencies, the central south regions show the least variation in hospital service efficiency averages, while the northern, southwestern, and northwestern regions exhibit significant changes, with the northeastern region experiencing the most substantial change at 52.89%. The central south regions, with their advanced medical service technology, higher economic development levels, and superior health resources, have their production technology sets at the optimal level, unlike other regions. The northern, southwestern, northwestern, and northeastern regions demonstrate more pronounced differences under the two frontier technology sets due to significant changes in the distance of the frontier, resulting in a decrease in the optimal level within each region, and the potential overestimation of the group frontier efficiency values (41). This also supports the validity of dividing the samples into groups in this study.

### 3.2 Analysis of the TGR in hospital service efficiency across various regions

Owing to the substantial disparities in resource endowments and geographical conditions across China’s regions, there is an inherent technological gap in hospital service efficiency. To quantitatively assess this gap, the TGR can be utilized. By applying the calculation from Equation 6, trends in TGR indicators for China’s six major regions from 2009 to 2022 can be observed.

**Figure 2:**
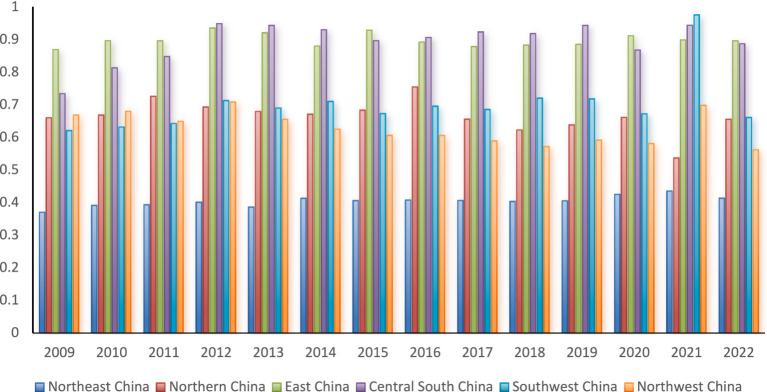
Evolutionary trends of TGR in the six regions.

As depicted in Figure 2, throughout the entire sample period, the TGR values of China’s regions can be categorized into three levels. The first level includes the East China and Central South regions, the second level consists of North China, Southwest China, and Northwest China, and the third level is the Northeast region. Furthermore, the TGR disparity between the first-level regions and the others is increasingly widening, a trend that aligns with findings from other research (3). Specifically, within the first level, the Central South and East China regions alternate in leading positions, with significantly higher TGR values than the other four regions, averaging 0.8951. This indicates that the hospital service efficiency in the Central South and East China regions is the highest, achieving over 89% of the potential optimal hospital service efficiency. These regions represent the pinnacle of hospital service efficiency in China, excelling in both technological and managerial aspects.

In the second level, the average TGR value is 0.6641, indicating a 33.59% improvement gap remains to reach the potential optimal hospital service efficiency in China. The Northeast region, which demonstrates the lowest hospital service efficiency, has an average TGR value of only 0.4043.

Several factors contribute to the higher TGR in various regions within the second tier compared to the Northeast. Firstly, the Western Development Strategy initiated in the late 20th century significantly boosted economic and social development in China’s western regions, particularly in the Southwest and Northwest. This strategy led to increased investment and a steady rise in health human capital, along with economic structural adjustments that greatly enhanced the medical and healthcare system. Secondly, despite facing economic challenges, North China benefits from vibrant areas like Beijing and Tianjin. These regions continue to drive growth in their surroundings and mitigate the gravitational pull of more prosperous areas like East China and Central South China.

Conversely, while the Northeast region has received national policy support, it struggles with a persistent economic slowdown and brain drain. The region’s lack of sufficiently strong core cities exacerbates these issues, leading to a notable disparity in healthcare efficiency compared to other regions.

### 3.3 Analysis of inefficiencies in hospital services

#### 3.3.1 National inefficiency analysis

Further analysis of the fundamental factors contributing to the inefficiency of regional hospital services can provide valuable insights for policymakers in developing targeted strategies for healthcare resource allocation and enhancing hospital service efficiency. Utilizing formulas (8) to (9), this study breaks down the inefficiency of hospital services at the joint frontier, denoted as MGEI, into two components: TGI and GMI. It then assesses the contribution of each to MGEI to identify the primary source of inefficiency in regional hospital services.

**Figure 3:**
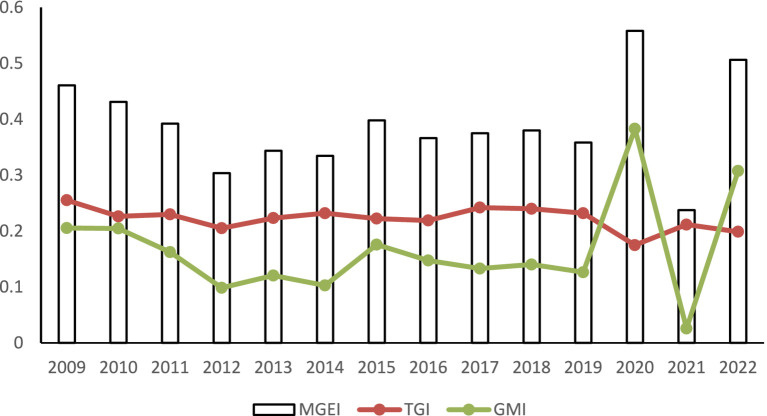
National hospital service inefficiency changes and trends.

Figure 3 illustrates the trends of MGEI and its two major components, TGI and GMI, at the national level between 2009 and 2022. As shown in Figure 3, from 2009 to 2022, with the exception of the epidemic period from 2019 to 2022, both China’s MGEI and TGI demonstrated a consistent downward trend, decreasing from 0.4606 to 0.3583 and from 0.2551 to 0.2319, respectively. Conversely, GMI exhibited a fluctuating downward pattern, declining from 0.2054 to 0.1263. This is attributed to the continuous reforms in China’s medical system since 2009, which involved multiple adjustments in management concepts, resulting in an overall fluctuating downward trend (10). The rapid changes in 2020 and 2022 may be associated with the onset and conclusion of China’s epidemic management, leading to subsequent disorder in the healthcare system due to the lack of relevant management experience. In 2021, the surge in medical demand during the epidemic likely played a role, concentrated and erupted in a year of relative stability in epidemic management and control.

The discrepancy between TGI and GMI suggests that the primary driver of improved hospital service efficiency in China is enhanced management within the healthcare system. However, the disparity in technical capabilities remains the most significant barrier to the efficiency of hospital health services across China’s regions.

#### 3.3.2 Analysis of the gap between TGI and GMI in various regions

**Figure 4:**
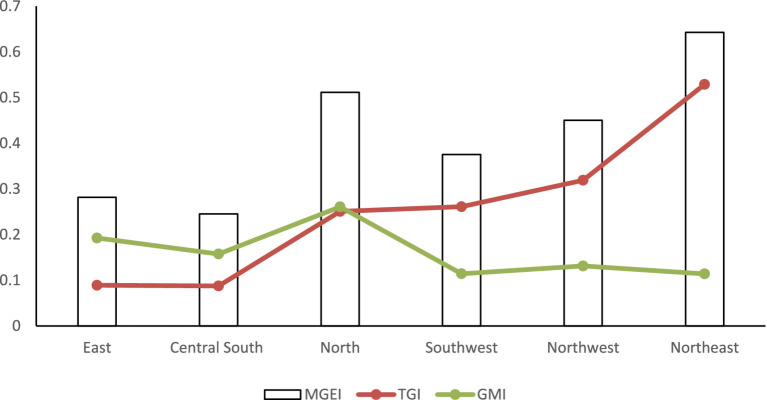
Decomposition of hospital service inefficiency in six major regions (2009–2022).

From a regional perspective, as depicted in Figure 4, the distribution of the TGI and the GMI reveals notable patterns. In more economically developed regions, such as East China, North China, and Southeast China, the GMI is greater than the TGI. In contrast, in less economically developed regions like Southwest China, Northwest China, and Northeast China, the TGI is higher than the GMI. This difference stems from the uneven economic development in China since the reform and opening-up period, during which a significant concentration of resources has been directed toward the eastern coastal areas, potentially at the expense of the development in central and western regions (42).

As a result, these western and northeastern regions face challenges such as insufficient investment in health fiscal funds, slow updating of health facilities, and low levels of health human capital. The difficulty in attracting foreign investment due to their remote geographic locations further exacerbates the significant disparity in TGI between the economically advantaged regions and the northwestern, southwestern, and northeastern areas. Meanwhile, management aspects can be more easily enhanced through inter-regional cooperation compared to technical aspects, leading to relatively lower GMI in the northwestern, southwestern, and northeastern regions.

For East and Central South China, the current priority should be the rational allocation of medical resources within the region. By doing so, these regions can promote overall efficiency improvements through enhanced resource utilization, ensuring that technological capabilities and management practices are optimized for better healthcare outcomes.

#### 3.3.3 Analysis of the gap between TGI and GMI in various provinces

**Figure 5:**
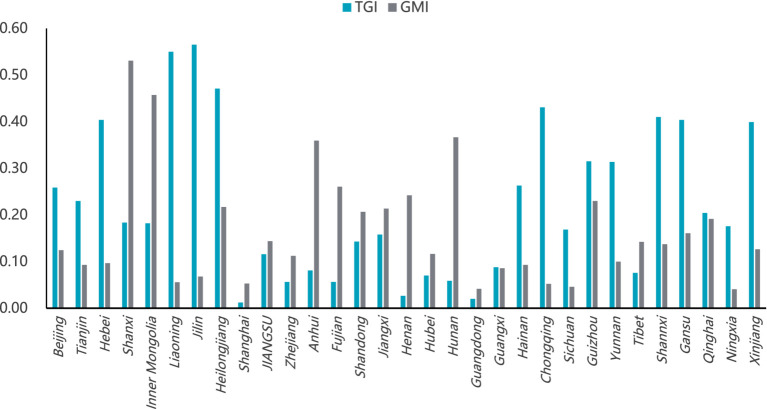
Decomposition of hospital service inefficiency by province (2009–2022).

The decomposition results of hospital service inefficiency and the proportion distribution of the two components for each province are shown in Figure 5. Taking Jilin in the Northeast region as an example, during the sample period, the mean value of its hospital service inefficiency at the common frontier was 0.6329, with TGI and GMI values of 0.5650 and 0.0679, respectively. In Jilin’s MGEI, TGI accounted for 89.2%, while GMI only accounted for 10.8%. This indicates that the primary reason for the inefficiency of hospital services in Jilin is that its technical equipment and technological innovation lag behind other regions, resulting in low overall efficiency. Therefore, it is necessary to introduce new equipment and technologies, strengthen technical ties with more advanced regions, and optimize the technical environment to improve hospital service efficiency.

In stark contrast to Jilin are the coastal provinces in East and Central South China, such as Shanghai, Zhejiang, and Guangdong. These regions benefit from superior geographical locations, favorable regional conditions, and higher levels of economic development, allowing them to rapidly introduce new equipment and technologies to enhance service efficiency. To further improve hospital service efficiency in these regions, a focus on management aspects is essential. This involves optimizing the allocation of medical resources within the region to establish a rational health resource distribution pattern. Additionally, enhancing the internal management capabilities of hospitals, including improving information and intelligent management systems, is crucial.

In most provinces and cities in regions like Northwest and Southwest China, where both technical conditions and management levels are low, improving service efficiency requires a dual approach. This involves not only introducing new equipment and technologies but also emphasizing the importation of hospital management experience from more developed areas to improve resource utilization efficiency. By doing so, these regions can achieve significant improvements in the efficiency of hospital health services.

### 3.4 Spatial effect analysis

#### 3.4.1 Spatial correlation test

Using the Global Moran’s I index, we assessed the hospital service efficiency of China’s 31 provinces with respect to 0–1 matrices (Table 4). The results show that the Moran’s I index for all years is positive and passes the significance test at the 5% level. This indicates that the efficiency of hospital services in China is not randomly distributed but exhibits spatial correlation and certain characteristics of spatial aggregation. These findings lay the foundation for further exploration of the spatial spillover effects between variables (43).

**Table 4:** The spatial correlation test results.

Year	Moran’s I	Z-value	*p*-value	Year	Moran’s I	Z-value	*p*-value
2009	0.218 **	2.127	0.017	2016	0.274 ***	2.554	0.005
2010	0.265 **	2.494	0.006	2017	0.353 ***	3.214	0.001
2011	0.172 **	1.706	0.044	2018	0.374 ***	3.391	0.000
2012	0.237 **	2.228	0.013	2019	0.229 **	2.177	0.015
2013	0.258 ***	2.417	0.008	2020	0.391 ***	3.561	0.000
2014	0.254 ***	2.382	0.009	2021	0.505 ***	4.46	0.000
2015	0.326 ***	3.004	0.001	2022	0.356 ***	3.252	0.001

*, **, and *** indicate statistical significance at the levels of 10, 5, and 1% levels.

#### 3.4.2 Spatial model test identification

Before conducting spatial panel regression, it is essential to perform a multicollinearity test of the variables. The results show that the VIF of the variables is 3.38, which is well below the empirical threshold value of 10, indicating that there is no multicollinearity issue among the variables. Next, this study follows the approach of Elhorst (44) to select an appropriate spatial econometric model. As shown in Table 5, the results from the LM and Robust LM tests suggest that the Spatial Durbin Model, which considers both the spatial error model and the spatial lag model, should be chosen. Additionally, the Hausman test results indicate that a fixed-effects model is appropriate. Finally, based on the LR test and Wald test results, the Spatial Durbin Model with double fixed effects is recommended (45).

**Table 5:** Model selection test results.

Test type	Value	*p*-value
LM-Lag test	182.91 ***	0.000
Robust LM-Lag test	4.74 **	0.029
LM-Error test	187.38 ***	0.000
Robust LM-Error test	9.23 ***	0.002
LR-Lag test	43.46 ***	0.000
LR-Error test	58.28 ***	0.000
Wald-Lag test	14.87 **	0.011
Wald-Error test	10.54 *	0.061
Hausman test	14.09 **	0.028

*, **, and *** indicate statistical significance at the levels of 10, 5, and 1% levels.

#### 3.4.3 Analysis of space overflow effects

Table 6 shows a significant positive spatial autoregressive coefficient for hospital service efficiency in China at the 1% significance level. This finding suggests that enhancements in hospital service efficiency in one area can positively influence the efficiency of hospitals in nearby regions. At the national level in China, the factors that most significantly contribute to hospital service efficiency are EDU, the LST, and ECON. All these factors positively affect hospital service efficiency. Conversely, factors such as LHE and OPEN have a negative impact on improving hospital service efficiency. Specifically, the coefficient for EDU is 9.933, with a non-significant spatial lag term, indicating that while human capital significantly boosts hospital service efficiency locally, its effect on neighboring regions is negligible. This could be due to the fact that a higher level of human capital provides vital talent for the advancement of regional healthcare, promoting the dissemination of advanced knowledge systems. However, the effect on neighboring areas is limited by the lagging impact of human capital (46) and the strong regional nature of employment in China’s medical sector, where local employment often provides more advantages, resulting in a limited diffusion of human capital. The coefficient for LST is 1.651, with a significantly positive spatial lag term, reflecting that technological development considerably enhances hospital service efficiency both locally and in neighboring regions. The coefficient for ECON is 0.009, with a negative but not significant spatial lag term, indicating that while economic development boosts local hospital service efficiency, its negative influence on neighboring regions is not substantial. This is possibly due to the interdependent relationship between hospital service efficiency and regional economies (15). Additionally, economically advanced areas often attract resources from weaker ones, a “siphoning effect” that can reduce the hospital service efficiency of surrounding regions. However, as regional economic disparities in China diminish, this effect is becoming less pronounced (47). The coefficient for LHE is −1.558, with a negative spatial lag term, suggesting that healthcare investment levels detrimentally affect hospital service efficiency within the same region. This is linked to inefficiencies in China’s healthcare sector, such as expanding services without considering efficiency or need (48), and imbalances in resource allocation across regions (49), which decrease overall healthcare efficiency. The coefficient for OPEN is −0.091, with a significantly positive spatial lag term, indicating that while openness has a non-significant effect on local hospital service efficiency, it significantly benefits neighboring regions. This is likely due to the variance in openness levels across China. In some areas, openness may be ineffective or even counterproductive (50). However, provinces like Shanghai and Guangdong effectively utilize foreign technology and capital, which improves healthcare service efficiency in neighboring areas.

**Table 6:** The result of Durbin models.

Variable	Coeff	z-value	*p*-value
EDU	9.933 ***	2.97	0.003
OPEN	−0.091	−1.22	0.224
LHE	−1.585 **	−0.23	0.026
LST	1.651 *	1.8	0.072
lnECON	0.009 *	1.86	0.063
ρ	0.109 *	1.65	0.098
Wx.EDU	−0.983	−0.15	0.882
Wx.OPEN	0.530 ***	4.6	0.000
Wx.LHE	−1.516	−1.02	0.308
Wx.LST	2.623 *	1.86	0.063
Wx.lnECON	−0.011	−1.09	0.276
Spatial rho	0.109 *	1.65	0.098
N	434		
Log-L	438.957		
Year effect	Yes		
Individual effect	Yes		

*, **, and *** indicate statistical significance at the levels of 10, 5, and 1% levels.

## 4 Discussion

### 4.1 Significant spatial variation in the efficiency of health services

The mean efficiency of hospital services in China is relatively low, averaging approximately 0.6 for the period 2009–2022. This finding contrasts with the study by Jiang et al. (17), which reported an efficiency score of approximately 0.8 using the Data Envelopment Analysis (DEA) model. The discrepancy arises from the DEA model’s omission of slack variables, which tends to inflate efficiency scores, whereas the Slacks-Based Measure (SBM) model incorporates these variables, resulting in lower efficiency scores. Significant spatial disparities in hospital service efficiency are evident across China’s six regions. A common frontier efficiency analysis reveals that efficiency is generally higher in the eastern and southern regions, and lower in the western and northern regions. This pattern diverges from the conclusions of Liu et al. (48) and Ye et al. (7). Liu et al. found the central regions of China to have the lowest health efficiency, while Ye argues that regional disparities in the efficiency of China’s health services are minimal. These differences can be attributed to the use of single-output models, such as Stochastic Frontier Analysis (SFA), by Liu and Ye, which may overlook variations in outputs arising from disparate social conditions and natural resource endowments across regions. From the perspective of the Technology Gap Ratio (TRG), there are also pronounced spatial differences in China’s regional technology gaps. East China and Central and South China lead, followed by Southwest China, Northwest China, North China, and Northeast China. This finding contrasts with Guo et al. (6), who reported a TRG order of East > Central > West. The primary reason for this discrepancy is the variation in regional division criteria, which can introduce errors in TRG measurement due to the overly broad geographic classifications. Therefore, to accurately assess the technology gap between regions, it is essential to consider comprehensively the geographic location, economic development level, and resource endowments, rather than simply dividing the provinces into eastern, central, and western regions.

### 4.2 Regional differences in managerial and technical inefficiencies

There is a more pronounced regional heterogeneity in hospital service inefficiency, with managerial inefficiency prevailing in East China and Central and South China, areas with relatively higher economic development. In contrast, technical inefficiency is more prevalent in Southwest, Northwest, and Northeast China. This pattern can be attributed to several factors. Firstly, the level of economic development in each region plays a crucial role. Regions with higher economic development tend to invest more in health technology, allowing them to maintain a technological edge. Conversely, in regions with lower economic development, the primary focus is often on meeting the population’s immediate health service needs, leading to lower prioritization of health technology advancements and consequently, slower technological progress. Secondly, China’s cross-regional health assistance model, which primarily involves targeted assistance to enhance the efficiency of health services in other regions, also influences these dynamics. This model helps optimize hospital operations by transferring organizational and management expertise. As a result, regions with lower economic development, such as the Southwest and Northwest, can achieve significant improvements in managerial efficiency.

## 5 Conclusion and suggestions

### 5.1 Conclusion

Using panel data from China’s 31 provinces between 2009 and 2022, this study conducts an empirical analysis of hospital service efficiency across six major regions, considering the diverse production technologies prevalent in different parts of China. Employing the common frontier theory, the study highlights variations in hospital service efficiency among provinces and regions, decomposes factors hindering efficiency, and uses the spatial Durbin model to investigate the spatial spillover effects of hospital service efficiency in China. The key findings are as follows:

First, due to uneven economic and social development, significant disparities exist among China’s provinces and regions regarding resources, economic power, and policy benefits. This results in varying efficiency frontiers for hospital services across different provinces. When compared against the national potential optimal hospital service efficiency, these gaps are pronounced. On average, the efficiency scores are: Central South China (0.7549), East China (0.7184), Southwest (0.6245), Northwest (0.5497), North China (0.4884), and Northeast (0.3571). Generally, the East and Central South regions exhibit significantly higher hospital service efficiency, forming the leading edge in China’s hospital service provision.

Second, the growth dynamics of China’s six major regions can be categorized into three levels: the first level includes the Central South and East regions; the second level comprises the Southwest, Northwest, and North regions; and the third level consists of the Northeast region. Notably, there is a general upward trend in growth dynamics across all regions, except for the Northwest. Between 2009 and 2021, the technological disparity between the East China and Central South regions and the rest of the country has widened, indicating significant technological barriers.

Third, the decomposition of inefficiency values shows that efficiency loss in the East China and Central South regions is primarily due to management inefficiency. In contrast, inefficiency in the Southwest, Northwest, and Northeast regions is mainly attributed to technical inefficiency. In North China, inefficiency stems from both technical and management inefficiencies.

Fourth, hospital service efficiency exhibits significant spatial spillover effects, meaning that the efficiency of hospital services in one province can positively influence the efficiency of neighboring provinces. The extent of these spatial spillovers varies and is influenced by several factors, including the availability of human capital, the level of openness to international influences, healthcare expenditure, the degree of technological innovation, and the stage of economic development.

### 5.2 Suggestions

To enhance the efficiency of healthcare services, it is imperative to precisely identify and address gaps in both technological and managerial domains. In regions such as East and Central South China, the strategic optimization of resource allocation, coupled with the avoidance of ineffective investments, should be prioritized. This can be achieved by fostering management innovation through competitive market dynamics. Furthermore, recognizing and leveraging spatial spillover effects is essential, requiring strengthened regional cooperation and the dismantling of technical barriers. This approach facilitates the diffusion of advanced technologies and management practices to less advantaged areas. Additionally, the formulation of long-term strategic plans that accommodate demographic shifts and evolving healthcare demands, alongside the enhancement of crisis management capabilities, is critical to ensuring the stability and sustainability of service efficiency (51).

## 6 Potential limitations and prospects

This study examines the trends, regional variability, and spatial spillover effects of hospital service efficiency across various regions in China from the perspective of regional differences. There are still areas that can be further refined and explored in future research.

Firstly, regarding the selection of research indicators, this study references the methodologies from existing literature. Future improvements can be made to enhance the scientific validity and replicability of the indicator system. Secondly, the traditional division into six regions may not fully capture the regional differences in China. Future research could consider more granular regional subdivisions, such as analyzing hospital service efficiency differences from the perspective of urban agglomerations. Third, this study’s analysis of spatial effects utilizes a single spatial weight matrix. Future research could explore the use of multiple matrices to provide a more comprehensive understanding of spatial dynamics.

## Data Availability

The original contributions presented in the study are included in the article/Supplementary material, further inquiries can be directed to the corresponding author.
